# Supplementary material for: Understanding the Impact of COVID-19 on Roma Vulnerable Communities in Western Romania: Insights and Predictive Factors from a Retrospective Study
Source: Viruses. 2024 Mar 12;16(3):435. doi: 10.3390/v16030435 (PMC10974346; doi:10.3390/v16030435)
Supplement: Supplementary file 1 [file viruses-16-00435-s001.zip › viruses-2890085-supplementary.pdf]

Supplementary material

Graphically, the ROC curves for each biomarker measured at hospital admission, in regards to ICU admission, can be observed in figures 1-7. Figure 8-14 show the ROC curves for each biomarker measured at hospital admission, in regards to the deceased status. Figure 15-21 show the ROC curves for each biomarker measured at ICU admission, in regards to the deceased status. The dot present on each line represents the cutoff value, calculated by the Youden index, which can be observed in the main manuscript (Table 4 for figures 1-14 and Table 7 for figures 8-14).

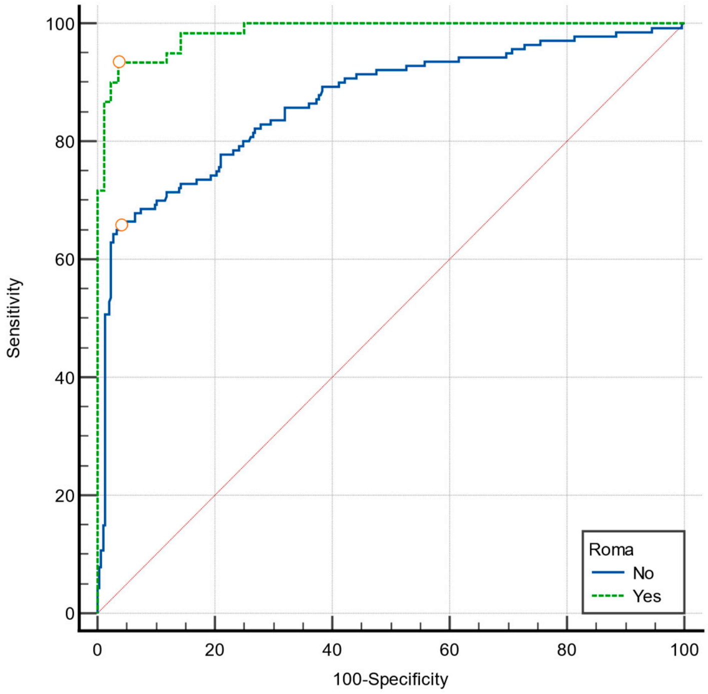

Figure S1. ROC curve analysis and comparison of CRP in regards to ICU admission, based on hospital admission data

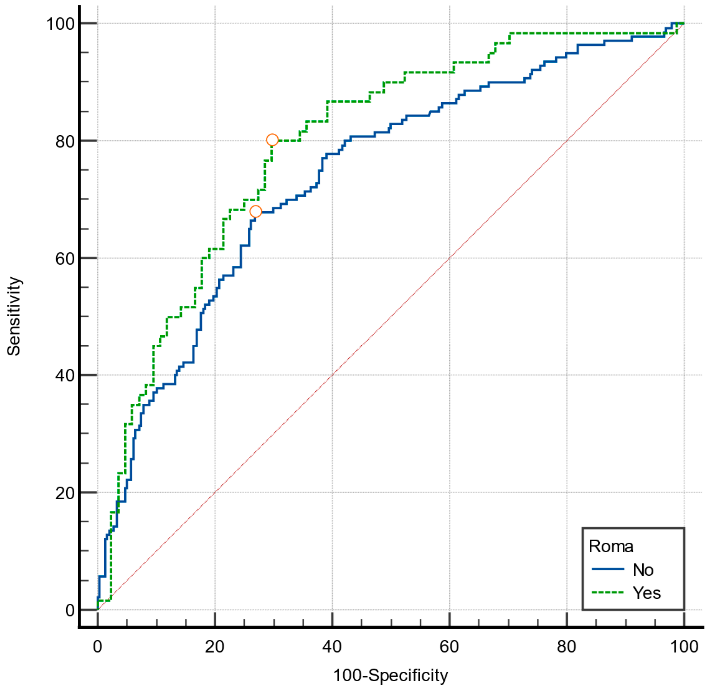

Figure S2. ROC curve analysis and comparison of ferritin in regards to ICU admission, based on hospital admission data

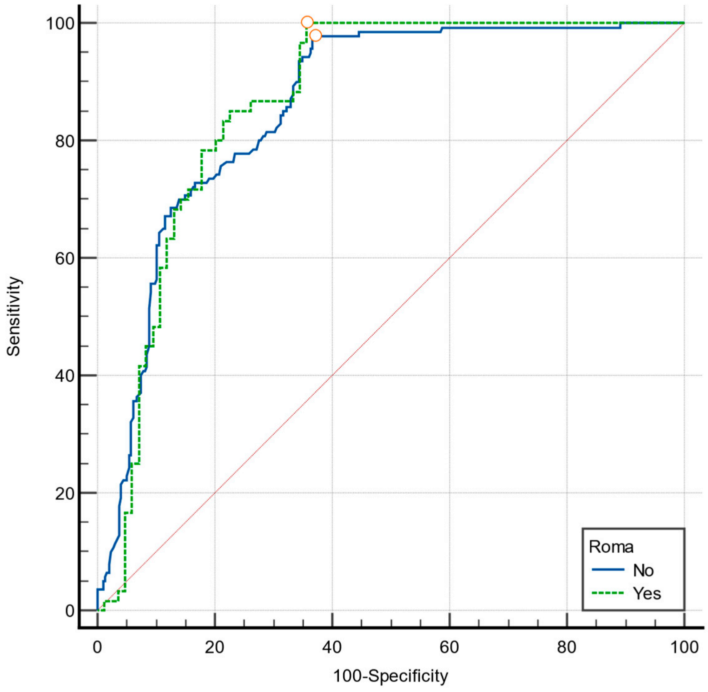

Figure S3. ROC curve analysis and comparison of IL-6 in regards to ICU admission, based on hospital admission data

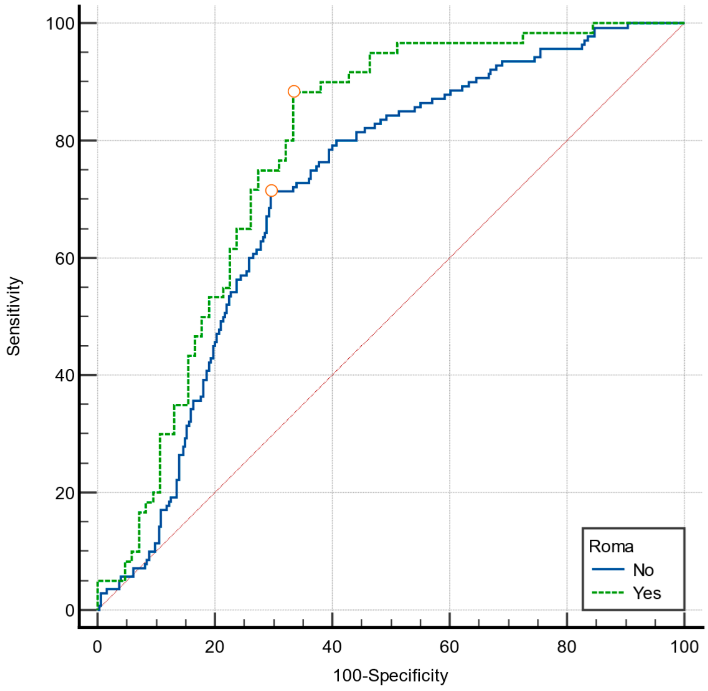

Figure S4. ROC curve analysis and comparison of D-dimers in regards to ICU admission, based on hospital admission data

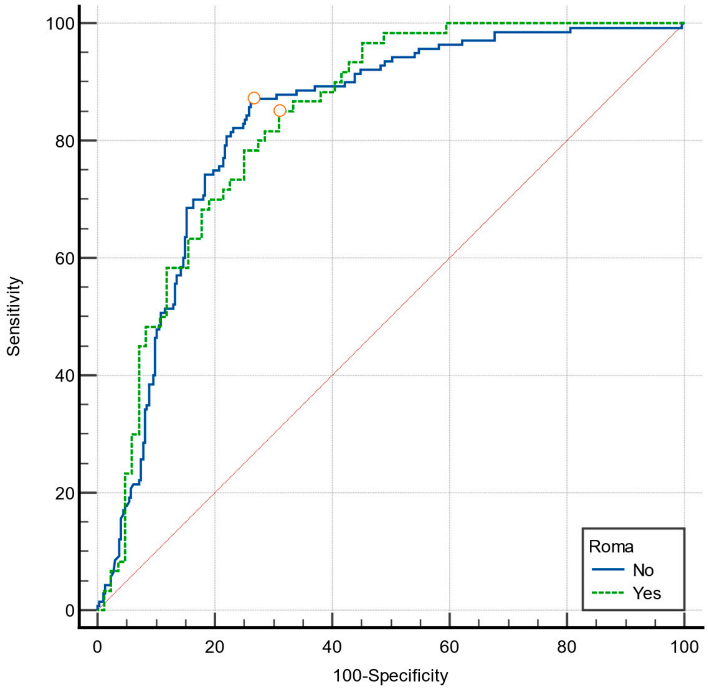

Figure S5. ROC curve analysis and comparison of LDH in regards to ICU admission, based on hospital admission data

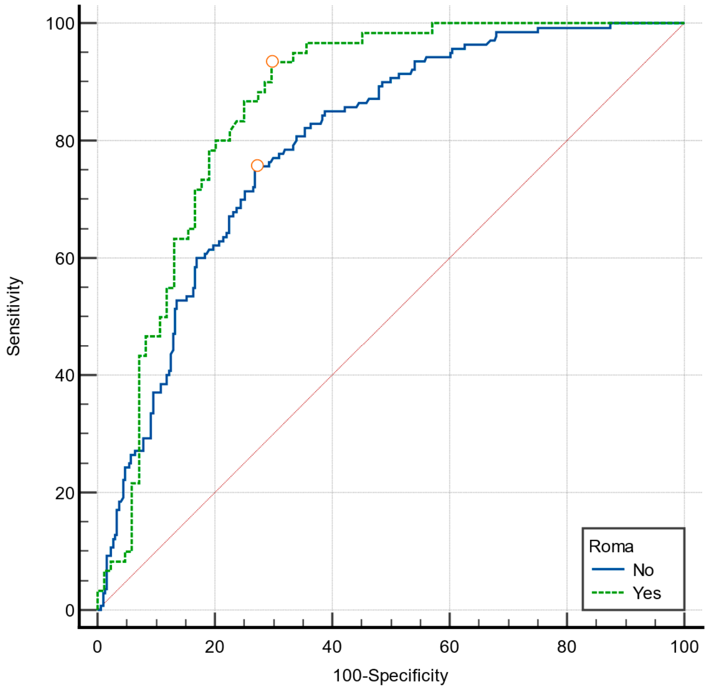

Figure S6. ROC curve analysis and comparison of HDL in regards to ICU admission, based on hospital admission data

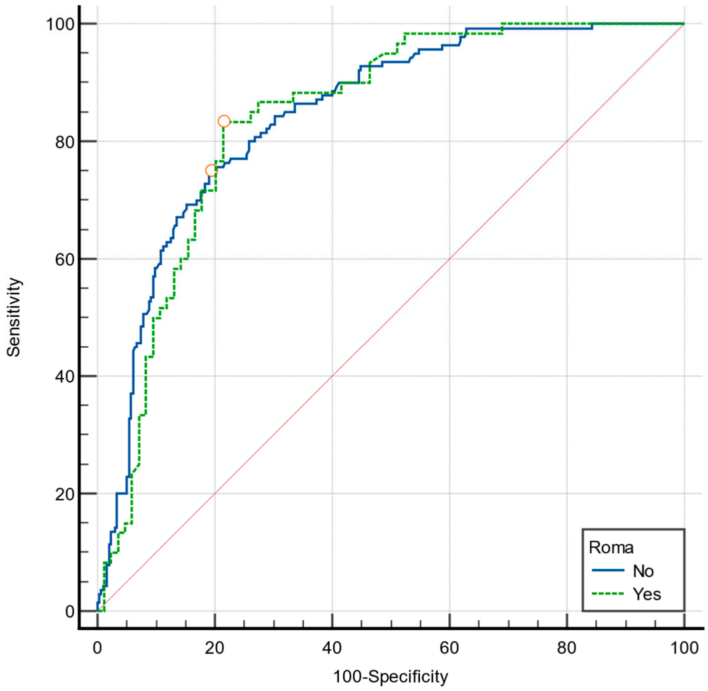

Figure S7. ROC curve analysis and comparison of 25-hidroxy-vitamin D in regards to ICU admission, based on hospital admission data

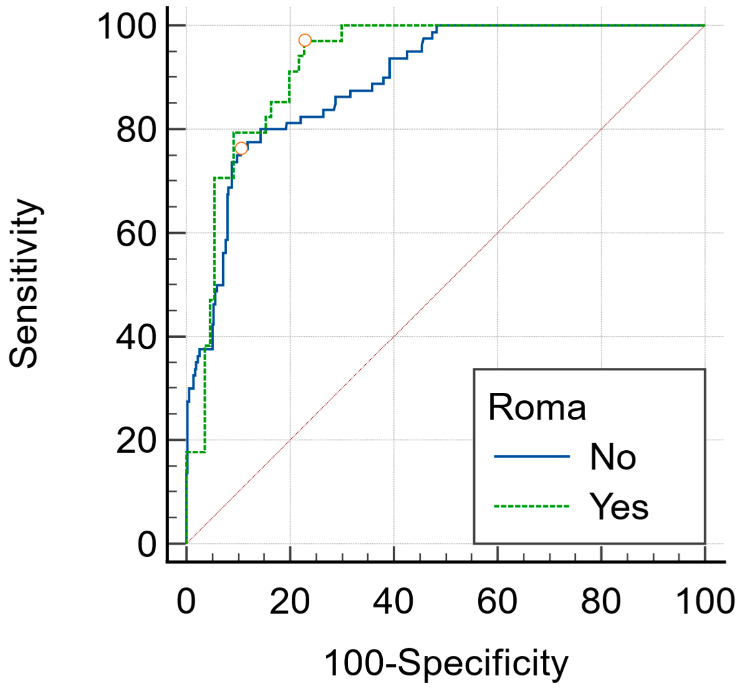

Figure S8. ROC curve analysis and comparison of CRP in regards to death, based on hospital admission data

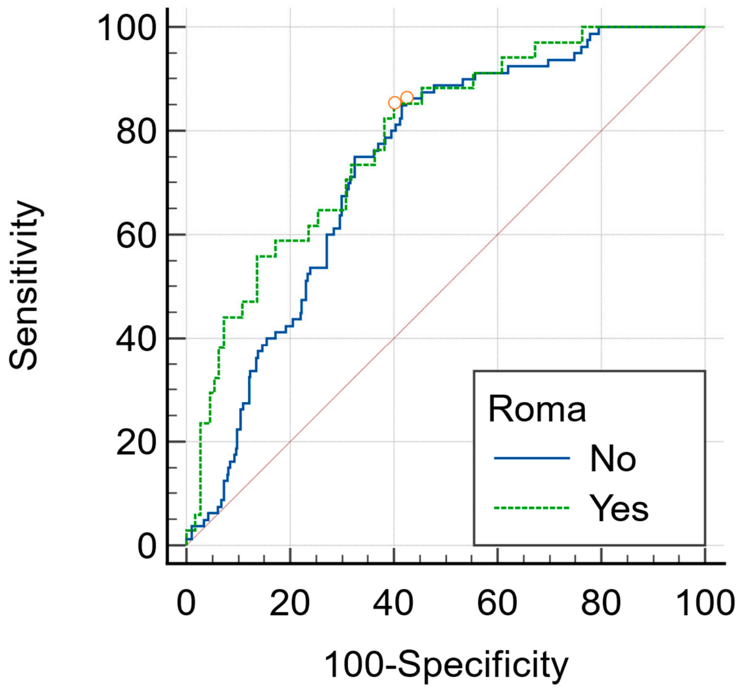

Figure S9. ROC curve analysis and comparison of ferritin in regards to death, based on hospital admission data

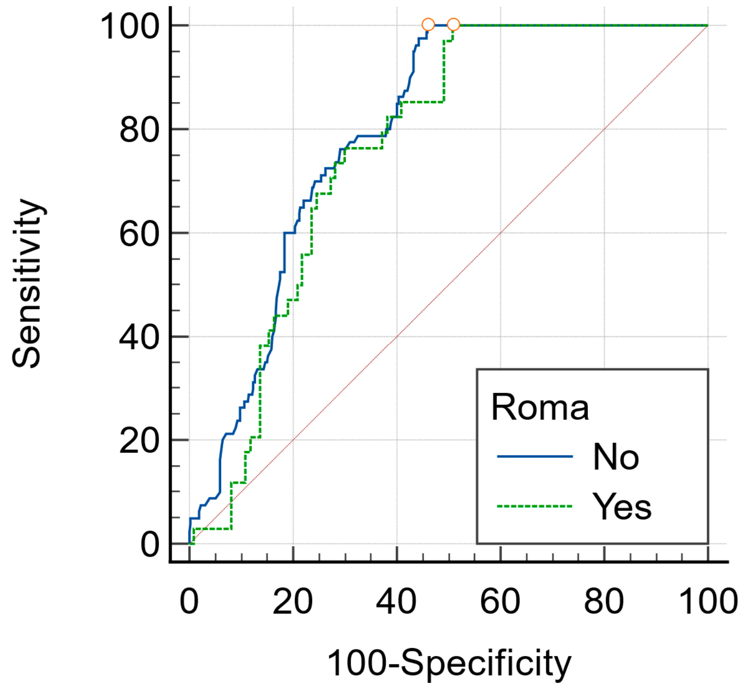

Figure S10. ROC curve analysis and comparison of IL-6 in regards to death, based on hospital admission data

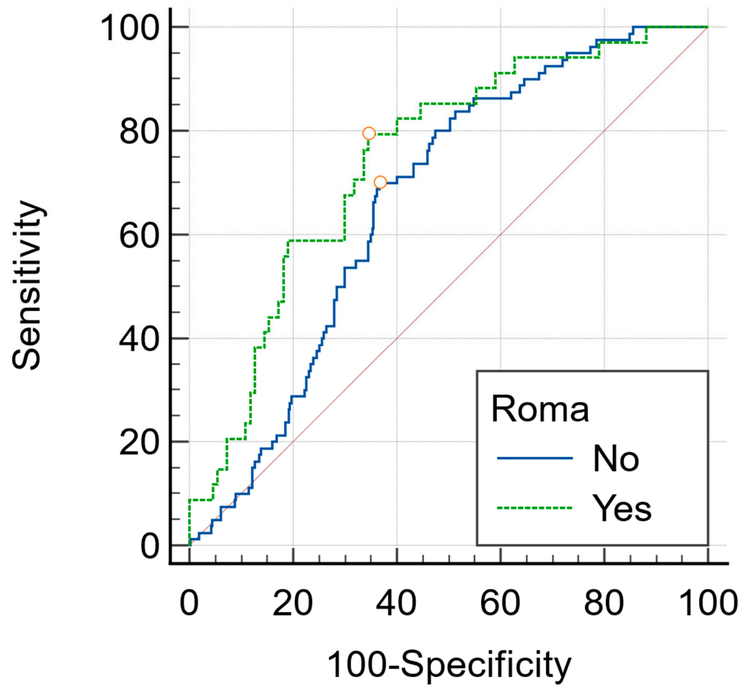

Figure S11. ROC curve analysis and comparison of D-dimers in regards to death, based on hospital admission data

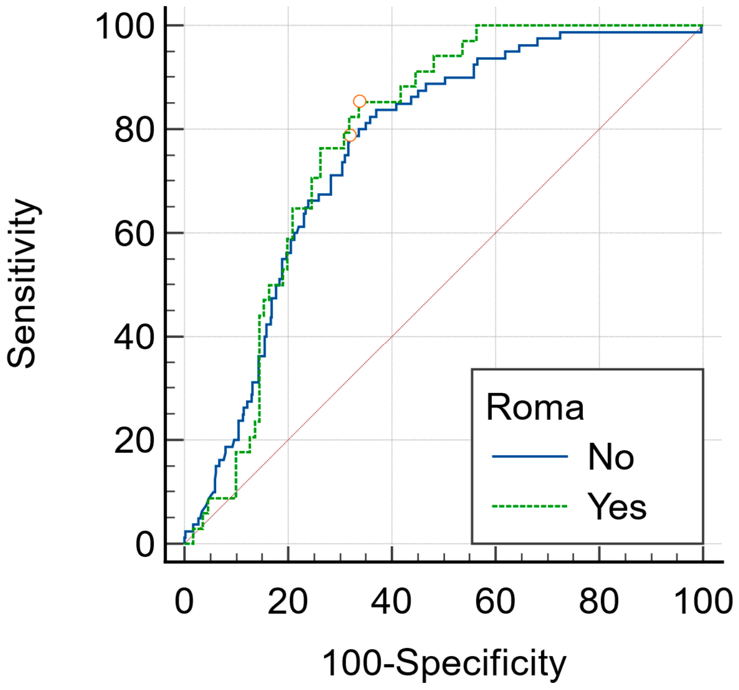

Figure S12. ROC curve analysis and comparison of LDH in regards to death, based on hospital admission data

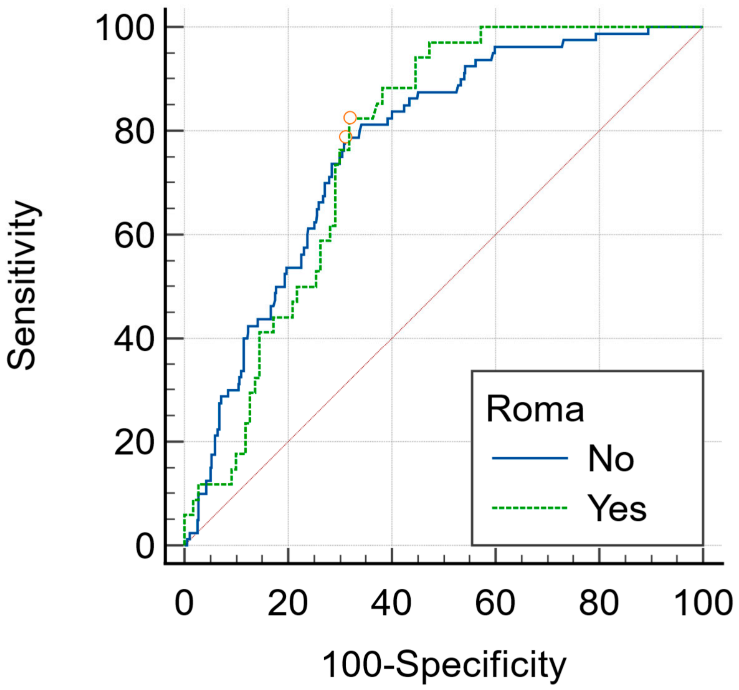

Figure S13. ROC curve analysis and comparison of HDL in regards to death, based on hospital admission data

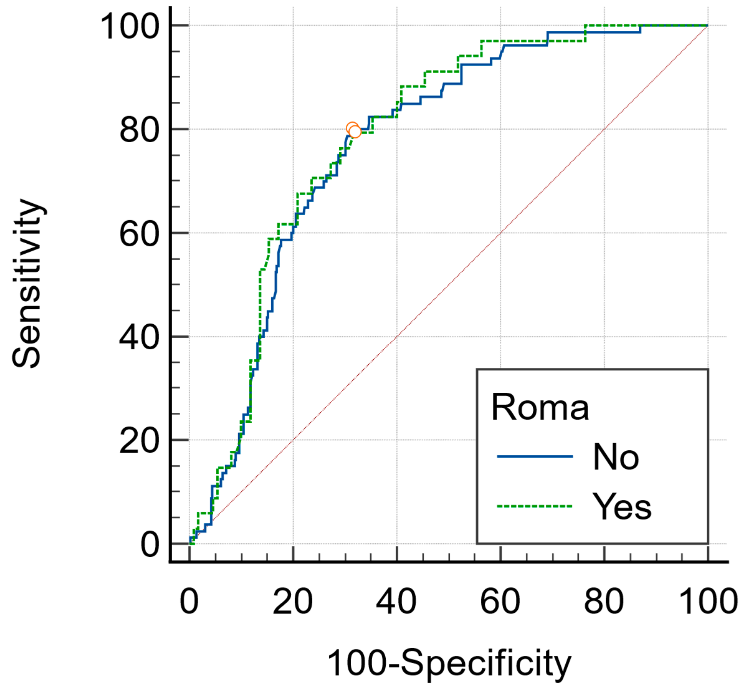

Figure S14. ROC curve analysis and comparison of 25-hidroxy-vitamin D in regards to death, based on hospital admission data

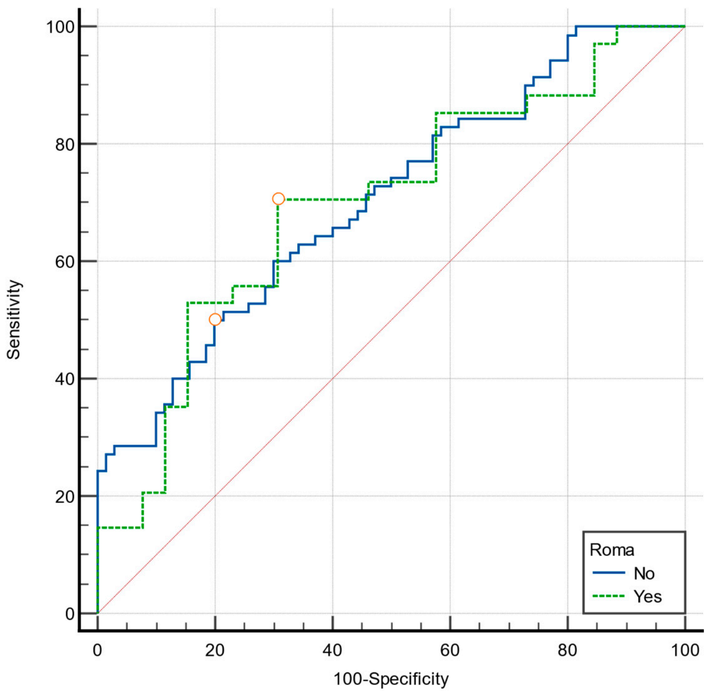

Figure S15. ROC curve analysis and comparison of CRP in regards to death, based on ICU admission data

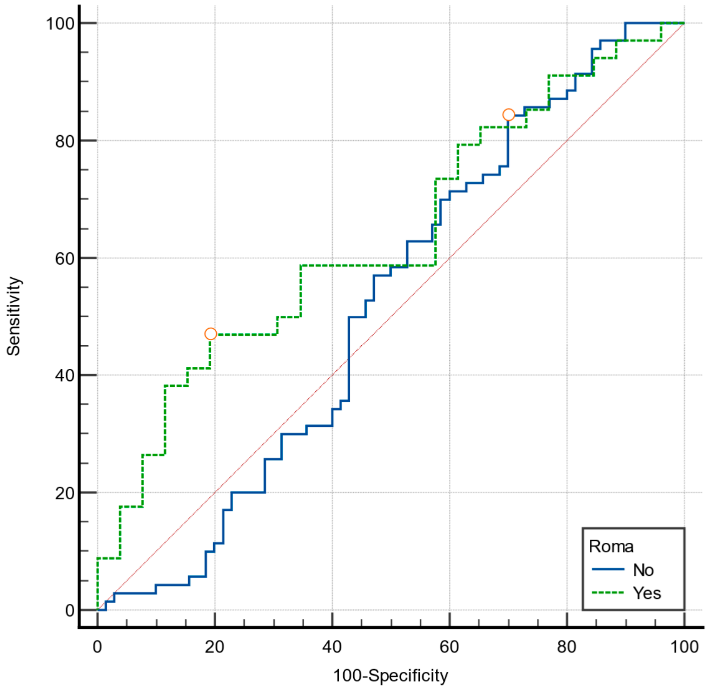

Figure S16. ROC curve analysis and comparison of ferritin in regards to death, based on ICU admission data

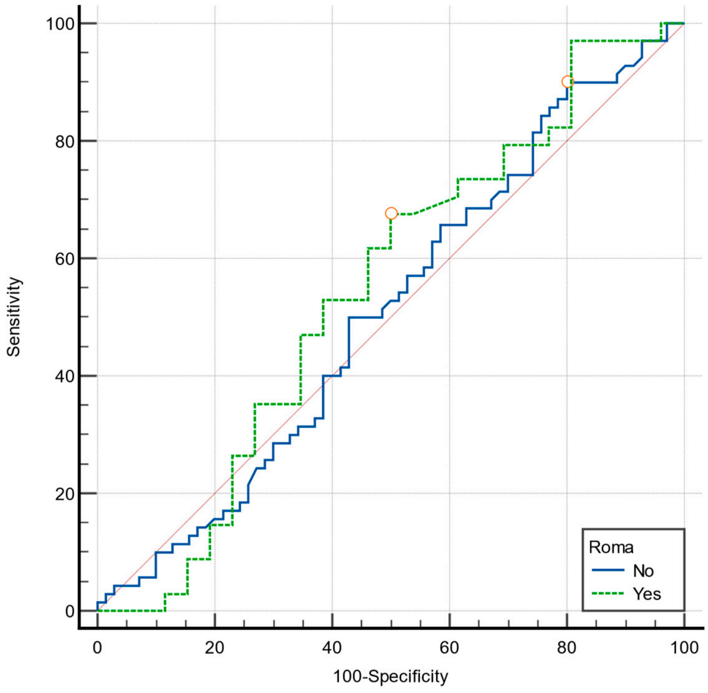

Figure S17. ROC curve analysis and comparison of IL-6 in regards to death, based on ICU admission data

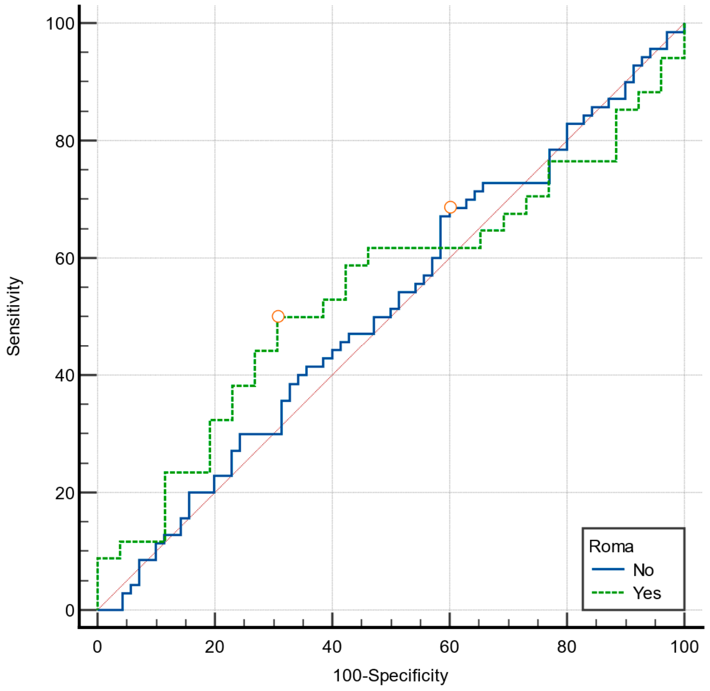

Figure S18. ROC curve analysis and comparison of D-dimers in regards to death, based on ICU admission data

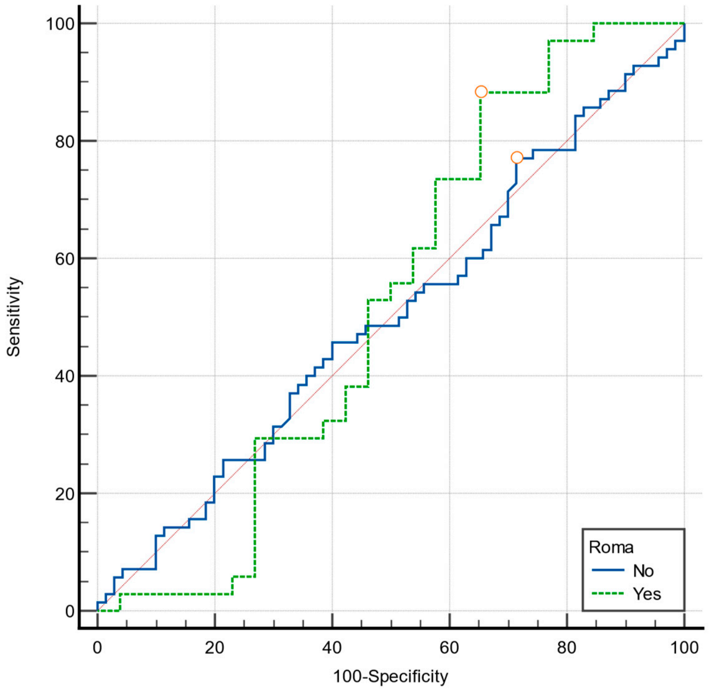

Figure S19. ROC curve analysis and comparison of LDH in regards to death, based on ICU admission data

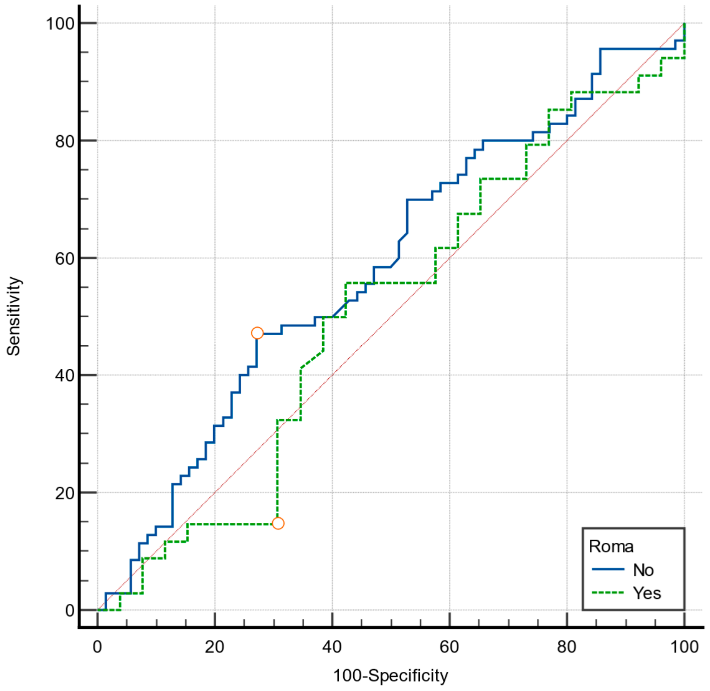

Figure S20. ROC curve analysis and comparison of HDL in regards to death, based on ICU admission data

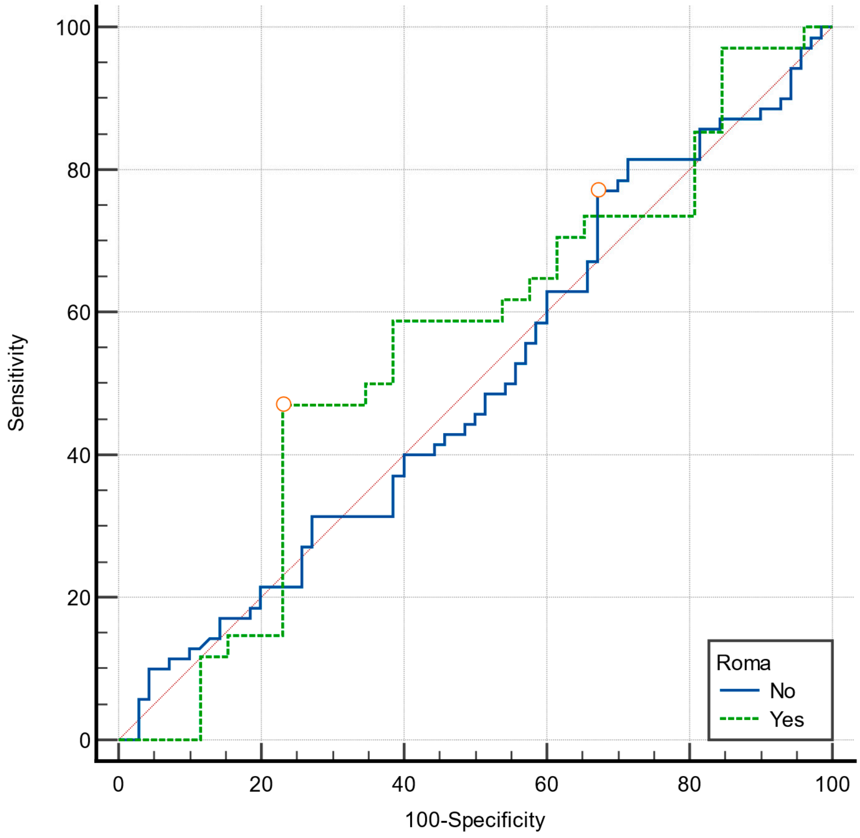

Figure S21. ROC curve analysis and comparison of 25-hidroxy-vitamin D in regards to death, based on ICU admission data
